# A case series to describe twelve fatal patients cause by rabies disease in central coast region, Vietnam in 2008

**Published:** 2011-01-10

**Authors:** Do Manh Hung, Le Van Tuan

**Affiliations:** 1 Pasteur Institute, Nha Trang City, Vietnam; 2 World Health Organization, Ho Chi Minh City, Vietnam

## Background

Human rabies fatal cases were related to animal rabies but it is reported that often lacks of animal rabies data, human case is underreported and rabies immune globulin and vaccine are not available so that these contributed to raise the big issues for rabies control and prevention measures in the developing countries. Recently in central coast region of Viet Nam human rabies fatal cases trend to increase from 1.5 to 2 folds in 2006 and 2007. This study described the clinical manifestations and epidemiological characteristics, post-exposure prophylaxis; and explored the risk factors associate with rabies fatality among twelve patients in central coast region in 2008.

## Methods

A case series descriptive study was used to reviewed medical records, conducted field investigation and interviewed household members of fatal case; And a case-control study was applied to explore the risk factors of rabies fatality.

## Results

Among 12 fatal cases, average age of fatal case was 20.9 years old and male is higher than female 2 times. Most of victims were pupil (66.7%). Average of incubation period is 97.4 days (20 - 332 days). 100% patient manifested typical symptom of furious rabies with aerophobia, hydrophobia, apprehension of light, excitability. Average time from furious spasm to death is 5.2 days (1 - 30 days). The majority of fatal cases received neither anti-sera (11/12 case) nor vaccination (10/12 cases). Domestic dog bite account for 100% of fatal case and the majority of them were unvaccinated (91.7%) and unleash (83.3%). 60% of local people did not know clean and flush wound with water and soap. Case-control study result showed that risk factors associated with rabies fatality are "Did not know vaccination for dogs is necessary"(OR= 33 (CI: 5.1 - 246.4) p<0.001); "Did not aware of signs and symptoms of animal rabies" (OR= 7.5, (CI: 1.1- 55), p<0.05); and "use suspected rabies animal for food" (OR=7.86; CI: 1.36-48.7; P<0.01).

## Conclusion

Rabies fatality must\can be prevented if we address to (1) enhance the active surveillance for animal rabies and vaccination domestic dog; (2) educate the school pupil, dog owner and local people on community prevention; (3) enhance the post-exposure prophylaxis, and anti-sera and vaccine should be available with a reasonable price at the district clinic\hospital.

